# Supplementary material for: Acceptability of Pharmacogenetic Testing among French Psychiatrists, a National Survey
Source: J Pers Med. 2021 May 21;11(6):446. doi: 10.3390/jpm11060446 (PMC8223981; doi:10.3390/jpm11060446)
Supplement: Supplementary file 1 [file jpm-11-00446-s001.zip › jpm-1219782-supplementary.pdf]

Supplementary materials

Table S1 : Survey questions

**Sociodemographic**
1.What is your gender? (male / female)
2.How old are you? (years)
3.What is the year of your specialized degree in psychiatry? (Before 1980 / 1980-1989 / 1990-1999 / 2000-2010 / 2010-2015 / 2015-2019 / 2020-2023)
4.What is your job status? (Resident / Assistant or University assistant / Hospital Practitioner / Professor)
5.In which department do you work? (department)
6.What is your main place of practice? (Psychiatric Hospital / University Hospital / General Hospital / Private practice / Other, specify)
7.What is your main practice area? (Adult Psychiatry / Child Psychiatry / Elderly Psychiatry / Addictology / Forensic Medicine / Other, specify)
8.What is your main theoretical orientation, clinical approach to psychiatry? (Neurobiological / Integrative / Psychoanalytical / Cognitivo-behavioral / Systemic / Other, specify)
**Perceived competency**
9.Have you ever prescribed pharmacogenetic tests? (yes / no)
10.Have you ever received any information on pharmacogenetics in your training, either initial or continuing? (yes / no)
11.Do you think you are informed enough to identify clinical situation in which testing is indicated? (yes absolutely / rather yes / rather no / not at all)
12.Do you think you are informed enough to explain to patients the risks and benefits of testing? (yes absolutely / rather yes / rather no / not at all)
13.Do you think you are able to adjust your therapeutic decision according to testing results? (yes absolutely / rather yes / rather no / not at all)
**Perceived usefulness**
14. Do you think a pharmacogenetic test might be useful to improve treatment response? (yes absolutely / rather yes / rather no / not at all)
15. Do you think that a pharmacogenetic test could be useful to improve treatment tolerability? (yes absolutely / rather yes / rather no / not at all)
16. Do you think that a pharmacogenetic test could be useful to assess the need for therapeutic dosage adjustment? (yes absolutely / rather yes / rather no / not at all)
17. Do you think a pharmacogenetic test could be useful to save time in patients' care? (yes absolutely / rather yes / rather no / not at all)
18. Do you think that the use of pharmacogenetic testing could become a common practice in psychiatry? (yes absolutely / rather yes / rather no / not at all)
**Intent to use**
19. Would you prescribe pharmacogenetic testing to guide your therapeutic decision in managing treatment-resistant depression? (yes absolutely / rather yes / rather no / not at all)
20. Would you prescribe pharmacogenetic testing to guide your therapeutic decision in the management of any depression? (yes absolutely / rather yes / rather no / not at all)
21. Would you prescribe pharmacogenetic testing to guide your therapeutic decision in the management of bipolar disorder? (yes absolutely / rather yes / rather no / not at all)
22. Would you prescribe pharmacogenetic testing to guide your therapeutic decision in the management of treatment-resistant schizophrenia? (yes absolutely / rather yes / rather no / not at all)

23. Would you prescribe pharmacogenetic testing to guide your therapeutic decision in the management of any schizophrenia? (yes absolutely / rather yes / rather no / not at all)
24. Would you prescribe pharmacogenetic testing to guide your therapeutic decision in the management of other psychiatric disorders? (yes absolutely / rather yes / rather no / not at all)
25. In a patient whose current treatment appears to be effective, would you change it to another more recommended treatment based on the results of pharmacogenetic testing? (yes absolutely / rather yes / rather no / not at all)
26. If you were a patient, do you think you would agree to receive a pharmacogenetic test from your doctor? (yes absolutely / rather yes / rather no / not at all)
**Reliability and ease of use**
27. Do you think that pharmacogenetic testing is easy to use? (yes absolutely / rather yes / rather no / not at all)
28. Do you think that pharmacogenetic testing is easy to access? (yes absolutely / rather yes / rather no / not at all)
29. Do you think that professional guidelines for the use of pharmacogenetic testing are clear? (yes absolutely / rather yes / rather no / not at all)
30. Do you think that the training and overall level of knowledge of physicians in pharmacogenetic is sufficient? (yes absolutely / rather yes / rather no / not at all)
31. Do you think the reliability and accuracy of these tests are sufficient? (yes absolutely / rather yes / rather no / not at all)
**Risks and limits**
32. Do you think that the cost of these tests could be a barrier to their use? (yes absolutely / rather yes / rather no / not at all)
33. Do you think that the waiting time before test results are available is a limit to its use? (yes absolutely / rather yes / rather no / not at all)
34. Do you think there is a risk of misuse of genetic data collected during pharmacogenetic testing (insurance, employment...)? (yes absolutely / rather yes / rather no / not at all)
35. Do you think there is a risk of incidental discovery of genetic diseases? (yes absolutely / rather yes / rather no / not at all)
36. Do you think there is a risk of psychological distress for the patient related to the test? (yes absolutely / rather yes / rather no / not at all)
37. Do you think that performing pharmacogenetic testing can negatively influence the therapeutic relationship? (yes absolutely / rather yes / rather no / not at all)
38. In the case of depressive disorder management, for example, do you think that the risk-benefit balance is generally in favor of performing a pharmacogenetic testing? (yes absolutely / rather yes / rather no / not at all)
39. Do you have any other concerns or worries about this tool? (yes / no)
Optional: If yes, what are they?
**Training**
40. Do you feel that you have received sufficient information and training on pharmacogenetics? (yes absolutely / rather yes / rather no / not at all)
41. Would you like to learn more about psychiatry applied pharmacogenetic? (yes absolutely / rather yes / rather no / not at all)
42. Optional: If Yes, what type of training would you like? (Several possible answers among: Inclusion in initial university training during residency / Specific university degree course / E-learning / Newspapers and journals / I don't know / Other)
